# Isolated Superior Mesenteric Artery Dissection: A Rare Etiology of Colic Ischemia

**DOI:** 10.7759/cureus.24819

**Published:** 2022-05-08

**Authors:** Kosisochukwu J Ezeh, Shannay E Bellamy

**Affiliations:** 1 Internal Medicine, Jersey City Medical Center, Jersey City, USA

**Keywords:** superior mesenteric artery, dissection, vascular, anticoagulation, acute abdomen, colic ischemia, sma

## Abstract

Although isolated superior mesenteric artery dissection is a rare disease entity, it possesses high mortality. The pathophysiology remains a mystery. In this case, we report a 68-year-old male with compromised coronary circulation been evaluated for coronary artery bypass surgery (CABG) and who developed a sudden, localized, right-sided abdominal pain. It was diagnosed on a computer tomography arteriogram (CTA), revealing a short segmental dissection involving the superior mesenteric artery (SMA). Vascular surgery was consulted and the decision was made to conservatively manage this patient's condition with anticoagulation. He was seen subsequently in the outpatient setting with a resolution of abdominal pain and a repeat computer tomography scan of the abdomen revealed resolution of previously seen colitis changes. Abdominal discomfort is a common complaint for which patients are seen in a variety of therapeutic settings, it is critical to bring attention to this case in order to raise awareness of the possibility of isolated SMA dissection as one of the underlying causes.

## Introduction

The term artery dissection refers to a tear in the arterial wall that results in a false lumen. Isolated superior mesenteric artery dissection is an extremely rare pathology. Its presentation is variable, ranging from asymptomatic incidental findings to an acute abdomen as bowel ischemia [1]. Certain risk factors have been described in association with SMA dissection such as hypertension, vasculitis, connective tissue disorder, atherosclerosis, trauma to the aorta, or, in some cases, no risk factors [2-3]. In our case, a 68-year-old male complaining of sudden right-sided pain and hematochezia with isolated superior mesenteric artery dissection was diagnosed on computer tomography. Treatment options vary from conservative with anti-coagulation to endovascular stenting or even surgery [1,4]. SMA dissection, given its anatomy distribution and involving the small bowel, requires immediate treatment and cannot be missed, as it is a potentially life-threatening diagnosis.

## Case presentation

A 68-year-old male with a past medical history of hypertension, coronary artery disease, hyperlipidemia, heart failure with reduced ejection fraction, diabetes mellitus, asthma, chronic obstructive pulmonary disease, benign prostatic hyperplasia, and anxiety disorder presented with complaints of generalized weakness and intermittent chest pain. He was being evaluated with plans for coronary bypass graft surgery following a CT- coronary that was remarkable for LAD occlusion when he developed right-sided abdominal pain, an episode of hematochezia, and hypotension during his hospitalization. He was treated conservatively with resuscitative measures. CT abdomen was conducted, showing colitis with extensive thickening of the right colon to the proximal transverse colon. His last colonoscopy was eight years ago, which was unremarkable.

**Figure 1:**
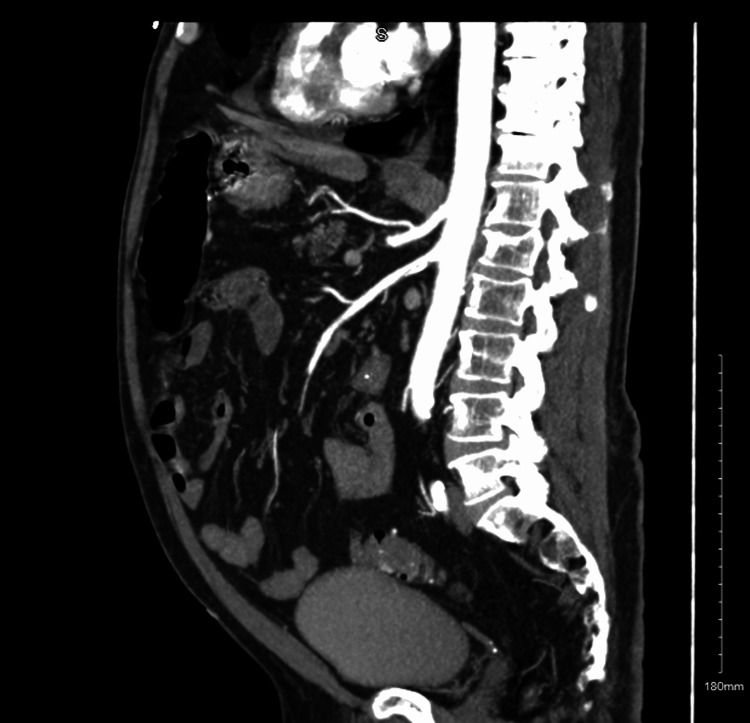
Showing marked the segmental area of dissection

Given the distribution of the pathology, CT angiography was ordered, which confirmed a short segmental area of dissection of the superior mesenteric artery, with extensive colitis of the right colon and hepatic flexure, as seen in Figure 1.

Vascular surgery was emergently consulted. Through the course of hospitalization, lactic acid remained within the normal range. The patient maintained hemodynamic stability following initial resuscitative measures. With an intravenous heparin drip, the patient was treated conservatively. There was no evidence of dissection propagation in the patient.

He was subsequently converted to oral anticoagulation and discharged home with close follow-up. A repeat CT abdomen revealed resolution of pathology.

## Discussion

Colic ischemia, commonly known as ischemic colitis, is a disorder in which blood supply to the colon is reduced (i.e., large bowel or large intestine). The term colic ischemia is generally accepted, as the inflammatory phase is not always present. Depending on the cause, symptoms might range from minor to severe. Colic ischemia is caused by an abrupt, although typically brief, decrease in blood flow, which is most noticeable in "the watershed" portions of the colon, where collateral blood flow is restricted. The superior mesenteric artery (SMA), inferior mesenteric artery (IMA), and internal iliac arteries supply blood to the large intestine and rectum. The treatment's goals are to restore blood circulation to the colon and preserve healthy digestive function. Treatment options for colic ischemia vary depending on the cause and severity of the damage.

Superior mesenteric artery (SMA) is an unfamiliar kind of arterial dissection that was first reported in 1947 [5]. It could either be isolated (spontaneous) or as an extension of aortic dissection. Although, among visceral arteries, SMA is the most common type of dissection [6]. It is more common in people aged 50-70 with a preference for the male gender [7]. It is seen in association with atherosclerosis, medial cystic necrosis, hypertension, vasculitis, fibromuscular dysplasia, and trauma [7]. In an autopsy series, the incidence of spontaneous isolated SMA dissection was 0.06% [8]. Clinical symptoms vary from acute severe abdominal pain and vague abdominal pain to asymptomatic [9].

Given how rare SMA dissection is, no standard therapeutic approach has been established. Current management options are separated into the surgical, conservative, and endovascular approaches [10]. The symptoms that the patient experiences guide the decision on how to proceed with treatment. There was no evidence of intestinal ischemia or rupture of any of the SMA branches in the patient like the one described in this case [11]. Conservative management is frequently used in situations like these. In cases such as ours, conservative management was employed. Patients are usually started on anticoagulation such as heparin drip, and repeat images are carried out to ensure no further dissection has occurred [12]. Patients are discharged if repeat imaging reveals no changes on oral anticoagulation and discharged home with close follow-up and vascular surgery.

## Conclusions

As demonstrated in our case, patients with abrupt abdominal discomfort are still prime candidates for superior mesenteric artery dissection. With technological advances in CT, practitioners can diagnose and halt potentially lethal disease propagation. Patients with manageable symptoms and no evidence of intestinal ischemia or injury to arteries to the dissection are typically treated conservatively with a heparin drip and switched to oral anticoagulants with close outpatient follow-up.

Case reports of SMA dissection, although they have been reported, still remain a rare lethal pathology, as it can involve the mesentery given its anatomical tributaries and present as a surgical emergency. While isolated SMA dissection is a rare condition, it has a substantial risk of morbidity and mortality. Therefore, it is a critical diagnosis to make.
